# The Genetic Spectrum of Maturity-Onset Diabetes of the Young (MODY) in Qatar, a Population-Based Study

**DOI:** 10.3390/ijms24010130

**Published:** 2022-12-21

**Authors:** Asma A. Elashi, Salman M. Toor, Ilhame Diboun, Yasser Al-Sarraj, Shahrad Taheri, Karsten Suhre, Abdul Badi Abou-Samra, Omar M. E. Albagha

**Affiliations:** 1 College of Health and Life Sciences (CHLS), Hamad Bin Khalifa University (HBKU), Qatar Foundation (QF), Doha P.O. Box 34110, Qatar; 2 Medical and Population Genomics Lab, Sidra Medicine, Doha P.O. Box 26999, Qatar; 3 Qatar Genome Program (QGP), Qatar Foundation Research, Development and Innovation, Qatar Foundation (QF), Doha P.O. Box 5825, Qatar; 4 Qatar Metabolic Institute, Hamad Medical Corporation, Doha P.O. Box 3050, Qatar; 5 Bioinformatics Core, Weill Cornell Medicine-Qatar, Education City, Doha P.O. Box 24144, Qatar; 6 Department of Biophysics and Physiology, Weill Cornell Medicine, New York, NY 10065, USA; 7 Centre for Genomic and Experimental Medicine, Institute of Genetics and Cancer, University of Edinburgh, Edinburgh EH4 2XU, UK

**Keywords:** maturity-onset diabetes of the young, MODY, diabetes, *HNF1A*, *HNF4A*, *GCK*

## Abstract

Maturity-onset diabetes of the young (MODY) is a rare monogenic form of diabetes mellitus. In this study, we estimated the prevalence and genetic spectrum of MODY in the Middle Eastern population of Qatar using whole-genome sequencing (WGS) of 14,364 subjects from the population-based Qatar biobank (QBB) cohort. We focused our investigations on 14 previously identified genes ascribed to the cause of MODY and two potentially novel MODY-causing genes, *RFX6* and *NKX6-1*. Genetic variations within the 16 MODY-related genes were assessed for their pathogenicity to identify disease-causing mutations. Analysis of QBB phenotype data revealed 72 subjects (0.5%) with type 1 diabetes, 2915 subjects (20.3%) with type 2 diabetes and 11,377 (79.2%) without diabetes. We identified 22 mutations in 67 subjects that were previously reported in the Human Genetic Mutation Database (HGMD) as disease-causing (DM) or likely disease causing (DM?) for MODY. We also identified 28 potentially novel MODY-causing mutations, predicted to be among the top 1% most deleterious mutations in the human genome, which showed complete (100%) disease penetrance in 34 subjects. Overall, we estimated that MODY accounts for around 2.2–3.4% of diabetes patients in Qatar. This is the first population-based study to determine the genetic spectrum and estimate the prevalence of MODY in the Middle East. Further research to characterize the newly identified mutations is warranted.

## 1. Introduction

Maturity-onset diabetes of the young (MODY) is a rare monogenic form of diabetes mellitus, accounting for approximately 1–5% of diabetes cases worldwide [1]. MODY is an autosomal dominant condition, clinically characterized by a multigenerational family history of diabetes and absence of pancreatic β-cell autoantibodies, and disease onset before the age of 25 years in most cases. A prominent feature of MODY includes progressive β-cell dysfunction, while in some cases it could be associated with other organ abnormalities, such as kidney abnormalities and fat malabsorption [2]. Patients with MODY are often misdiagnosed due to similarities in their clinical manifestations to those with other forms of diabetes [3]. To date, 14 genes are ascribed to the cause of MODY, each encoding for a different subtype [2]. Recent studies have identified novel variants in two genes encoding regulatory factor X6 (*RFX6*) and NK6 Homeobox 1 (*NKX6-1*) as possible MODY contributors [4,5].

MODY screening and profiling in clinic is challenging due to the absence of a single clinical criterion, the occurrence of de novo mutations and the unavailability of suitable genetic testing [6]. The genetic architecture of diabetes is complex. For instance, genetic variations in several MODY genes have been associated with type 2 diabetes mellitus (T2DM) in genome-wide association studies (GWAS) [7,8]. In addition, homozygous mutations in some MODY genes can cause other types of diabetes, such as permanent neonatal diabetes and antibody-negative type 1 diabetes mellitus (T1DM) caused by mutations in the insulin gene (*INS*; MODY10) [9,10]. Moreover, homozygous mutations in *ABCC8* (MODY12) and *KCNJ11* (MODY13) also cause neonatal diabetes [11]. Studies have shown that approximately 80% of MODY patients were misclassified as either T1DM or T2DM, leading to suboptimal therapeutic intervention [3]. Prior studies have, however, revealed certain unique phenotypic features that assist in the identification of suspected MODY patients, such as low apolipoprotein levels in MODY1, and renal cysts and diabetes syndrome in MODY5 [12,13,14]. In view of this, understanding the genetic architecture and physiological anomalies associated with MODY are essential and molecular genetic testing is crucial for identifying patients with MODY.

The prevalence of MODY varies amongst different populations [15]. The most frequent subtype in the United States, Japan and Turkey is *GCK*-MODY2, accounting for approximately 50% of the identified variants, followed by *HNF1A*-MODY3 and *HNF1B*-MODY5 [16,17,18,19,20]. In contrast, *HNF1A*-MODY3 is the most common MODY subtype among the studied populations in South Africa, South Korea and in Europe, including the United Kingdom, Germany and Poland [1,15,21,22]. MODY prevalence in the Middle East and North Africa (MENA) region remains largely unexplored [23]. Although there have been reports of MODY prevalence in Kuwait, Oman and Tunisia from suspected cases, a definitive population-based prevalence estimate has not been determined.

Pathogenic and likely pathogenic variants have been previously identified in 2.1% of pediatric diabetes patients [24]. Notably, a recent clinic-based study of MODY in children from Qatar has shown that MODY affects 4.56 children per 100,000 in Qatar [25]. However, this study focused primarily on children between the ages of 0 and 18 years and included children from different ethnicities attending a single hospital [25]. In this study, we estimate the prevalence and determine the genetic spectrum of MODY in Qatar using whole-genome sequencing (WGS) data from the population-based cohort of Qatar Biobank (QBB) participants (*n* = 14,364). We sought to determine the spectrum of MODY-related mutations and identify highly penetrant Qatari-specific mutations. Our findings provide important insights into the genetic architecture of MODY in the Middle East and have the potential for improving the diagnosis and clinical management of MODY patients in Qatar.

## 2. Results

### 2.1. Study Overview

**Figure 1:**
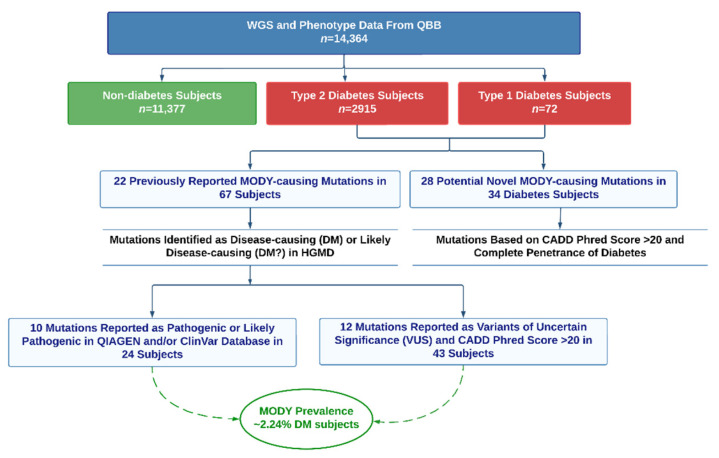
Study Design. This study was based on whole-genome sequencing (WGS) data of the Qatar Biobank (QBB) participants. The analysis cohort comprised *n* = 14,364 subjects, classified into diabetes mellitus subtypes (T1DM; *n* = 72 and T2DM; *n* = 2915) and non-diabetes subjects (*n* = 11,377) based on phenotypic data. The detected mutations were classified into previously known or potentially novel MODY-causing mutations based on pathogenicity annotations in the human gene mutation database (HGMD), QIAGEN Clinical Insight and ClinVar databases or Combined Annotation-Dependent Depletion (CADD) scores, as well as on disease (diabetes mellitus) penetrance.

The overview of the study design is illustrated in Figure 1. This study was based on WGS and phenotypic data of 14,364 participants from the population-based QBB cohort [26]. Analysis of phenotype data showed that 72 participants (0.5%) of the study cohort were classified as T1DM, 2915 (20.3%) as T2DM, while 11,377 (79.2%) were classified as non-diabetes. The clinical characteristics of the study population are listed in Table 1. Female participants constituted 55.8% of the study population, while the age of the overall study cohort ranged from 18 to 89 years. Notably, the prevalence of obesity in Qatar is remarkably high (77.6% of the total study population were overweight or obese). Additionally, 74.1% of non-diabetes participants and 91.0% of diabetes patients in our study were overweight or obese. Only 35 subjects (0.24%) met the clinical criteria for diagnosing MODY based on being diabetic with normal BMI and the age of onset before <25 (only 63% of diabetes patients declared age of disease onset). Therefore, the widely accepted clinical criteria for MODY diagnosis was not accurate when applied to our study cohort, given that most patients were overweight or obese, and genetic testing is critical in MODY diagnosis.

### 2.2. Identification of Previously Known MODY Causative Variants

We first investigated previously known variants associated with MODY onset. We investigated variants that were previously reported as disease-causing (DM) or likely disease-causing (DM?) mutations for MODY in the human gene mutation database (HGMD) and classified as pathogenic or likely pathogenic in Qiagen Clinical Insight (QCI) and/or ClinVar databases (Table 2). QCI classification criteria are based on the 2015/2016 ACMG/AMP Guidelines. Variants were also annotated by SIFT and PolyPhen-2 databases to indicate the potential functional effect of each mutation. We detected 10 previously identified missense MODY mutations in 24 participants (Table 2). The majority of detected variants were located in *HNF1A*. However, rs587778397 (p.Arg177Trp) in *HNF1A* and rs766934515 (p.Val113Gly) in *BLK* were the most frequently occurring mutations. Additional variants in *HNF4A*, *GCK*, *KLF11* and *ABCC8* were also recorded. Moreover, 10 subjects carried mutations in the *HNF1A* gene, 5 in *BLK* and 4 in *GCK*. Complete penetrance was observed in *HNF4A* (rs1555817727; p.Arg312Cys) and *HNF1A* (rs137853238; p.Arg272His).

In addition, we also identified variants of uncertain significance (VUS) that are predicted to rank in the top 1% of most deleterious mutations in the human genome based on CADD Phred scores (>20). We identified 12 missense VUS in 43 subjects (Table 3). Most subjects were heterozygous carriers of variants in *HNF1A* (*n* = 26), followed by *GCK* (*n* = 5) and *BLK* (*n* = 5) genes. *HNF1A* (rs202039659; p.His514Arg) was the most frequent mutation, recorded in 11 subjects. However, complete penetrance was only recorded for *HNF4A* (rs371124358; p.Arg310Gln and rs377151067; p.V404I). Combined, we found that previously known MODY mutations are mostly detected in the *HNF1A* gene, accounting for 53.7% of cases, followed by *BLK* and *GCK* mutations accounting for approximately 15% and 13% of cases, respectively. Overall, we identified 22 MODY-causing mutations in 8 genes, accounting for 2.2% of diabetes participants (Table 2 and Table 3).

### 2.3. Identifying Potentially Novel MODY-Causing Mutations

**Figure 2:**
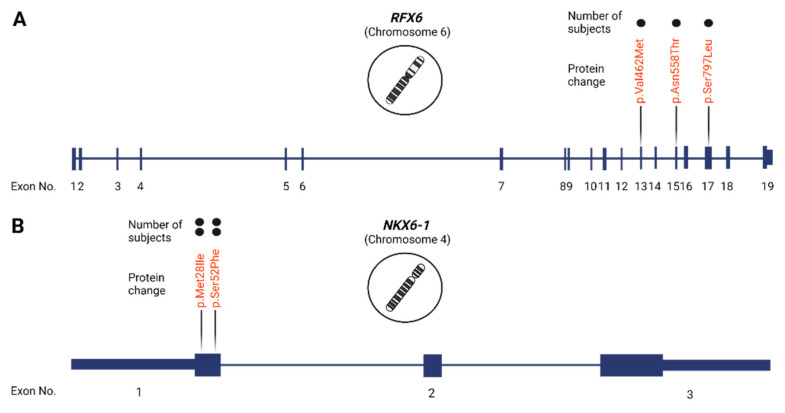
Identified mutations in *RFX6* and *NKX6-1*. The locations of potentially novel MODY-causing mutations in (**A**) *RFX6* and (**B**) *NKX6-1* genes. The number of participants carrying each mutation is presented by a dot above each gene mutation.

Next, we investigated potentially novel MODY-causing mutations based on complete disease penetrance (100%) and a CADD Phred score >20. We detected 28 mutations in 14 genes in 34 diabetes subjects (Table 4). None of these variants are reported as MODY-causing mutations in the HGMD or ClinVar databases, while the majority are predicted to be damaging by Polyphen-2 and deleterious by SIFT. In addition, 18 mutations are not reported in dbSNP or the Genome Aggregation Database (gnomAD). All detected novel mutations were missense, while variants in *HNF1A* (MODY3) and *CEL* (MODY8) were the most common, detected in eight subjects. Moreover, we also detected variants in rare MODY-related genes, including *NEUROD1* (MODY6), *KCNJ11* (MODY13) and *APPL1* (MODY14), among other MODY subtypes. Notably, we also identified three variants in *RFX6* and two variants in *NKX6-1* in seven subjects (Figure 2). Importantly, all potentially novel mutations were detected in patients classified as T2DM based on phenotype data.

### 2.4. Estimating the Prevalence of MODY in Qatar

**Figure 3:**
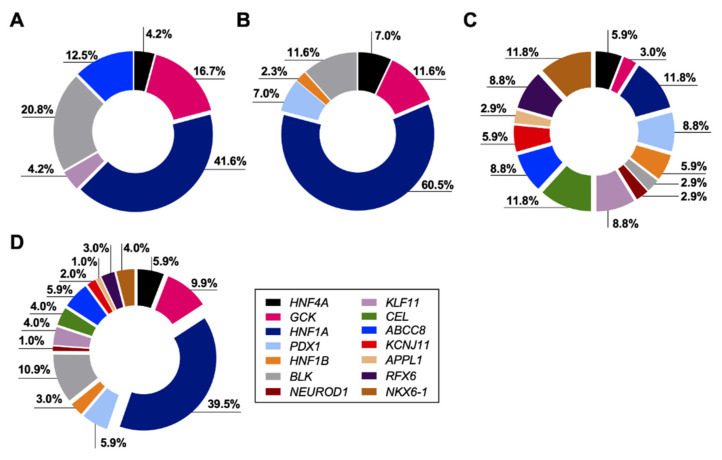
Proportion of subjects carrying MODY-causing mutations. Donut charts represent the proportions of subjects carrying: (**A**) previously reported pathogenic and likely pathogenic MODY-causing mutations (*n* = 28 subjects); (**B**) previously reported disease-causing (DM) or likely disease-causing (DM?) variants in the Human Gene Mutation Database, predicted to be among the top 1% most deleterious mutations in the human genome (*n* = 43 subjects); (**C**) novel fully penetrant, potentially MODY-causing mutations detected and predicted to be among the top 1% most deleterious mutations in the human genome (*n* = 34 subjects). (**D**) Donut chart illustrates the overall proportions of subjects (*n* = 101) carrying the previously known and potentially novel MODY-causing mutations. All genes are color coded, while percentages of subjects carrying each mutation subtype are also shown.

We detected 22 previously known MODY-causing mutations in 67 subjects, yielding a prevalence of MODY at 2.2% in individuals with diabetes (Figure 1). In addition, we identified 28 potentially novel MODY-causing mutations, accounting for ~56% of the overall reported mutations in 34 subjects and 1.14% of diabetes cases. Of note, combining subjects carrying mutations in both known and potentially novel MODY-causing mutations in our study population yielded an overall prevalence of 3.4% of diabetes patients in Qatar (Figure 1). Overall, previously reported and potentially novel MODY mutations were detected in 101 subjects. All detected mutations were heterozygous. The proportions of subjects carrying mutations in each MODY subtype are illustrated in Figure 3. We found that *HNF1A*-MODY3 is the most prevalent MODY subtype in the Qatari population, accounting for approximately 40% of cases. In addition, we also compared the clinical characteristics of subjects carrying MODY-related mutations (Supplementary Table S1). The identified MODY patients were predominately overweight and obese (83.1%), while they also showed strong diabetic medical history (95%). Moreover, the gender distribution of MODY cases showed higher prevalence in females (54.5%). In addition, we also compared the clinical parameters and genetic matrices of MODY-related genes between non-diabetes, T1DM and T2DM patients (Supplementary Table S2 and Supplementary Figure S1). Based on clinical parameters, half of the participants who carried mutations in MODY-related genes were classified as T2DM patients and half were non-diabetes based on phenotype data, while one patient was classified as T1DM (Supplementary Table S2). The genetic profile of these subjects also showed that the majority of them carried mutations in *HNF1A* (Supplementary Figure S1).

## 3. Discussion

The etiopathogenesis and clinical manifestations of MODY are broadly analogous to other forms of diabetes, such as youth-onset T2DM, which renders clinical risk assessment and genetic testing critical for accurate diagnosis and therapy selection. However, due to the challenges associated with genetic screening, MODY is underreported in the MENA region. In Europe, MODY accounts for 1–5% of all diabetes mellitus cases [28]. We estimated the prevalence of MODY in Qatar at 2.2% of diabetes patients, while inclusion of potentially novel mutations yielded a higher proportion of MODY, accounting for 3.4% of cases. The MENA region has one of the highest and fastest increasing levels of diabetes prevalence compared to global estimates [29]. We estimated the prevalence of diabetes in the population-based QBB cohort as ~21%, in agreement with previous reports (~17–20%) [30,31,32].

The prevalence of MODY mutations among children with diabetes from different ethnicities was estimated at 4.56 per 100,000 individuals (~1.5% of diabetes cases) according to a recent single clinic-based study conducted in Qatar [25]. Among the 12 mutations detected in children, 9 mutations were also detected in our study cohort. These included one previously reported mutation in *HNF1A* (rs202039659; p.His514Arg) and a previously known mutation in *GCK* (rs1375656631; p.Ala259Thr). However, seven additional mutations were detected in our study cohort but they did not meet our analysis criteria, such as HGMD and Clinvar classifications. Overall, the differences in reported variants among MODY cases in children compared to Qatari adults could be attributed to differences in study design, given that our study was a large population-based cohort of Qatari adults (18–89 years), whereas the previous study was a small study of children with different ethnicities attending a single diabetes clinic in Qatar.

We identified 22 previously reported and 28 potentially novel MODY-causing mutations in 101 participants, which accounted for 3.4% of diabetes patients. The estimated prevalence is in accordance with the widely accepted overall prevalence of MODY, accounting for 1–5% of all diabetes cases. Combined, mutations in *HNF1A* were the most common, detected in 40 cases (39.6%); followed by *BLK* (10.9%) and *GCK* (9.9%) genes. *HNF1A* MODY-causing mutations are the most prevalent in European, North American, and Asian populations; accounting for 20–50% of all MODY cases [3,6,33,34,35]. We also detected several known but rare MODY-causative mutations in different genes including *PDX1*-MODY4, *KLF11*-MODY7 and *ABBC8*-MODY12. However, many subjects carrying previously reported mutations did not present the clinical symptoms of diabetes, which could be attributed to incomplete penetrance of MODY mutations, which varies with differences in age and in genetic architecture between populations [6,36]. MODY is well known for its clinical and genetic heterogeneity with variable disease penetrance. Notably, the associations between *BLK* mutations and MODY penetrance has been indecisive. Five MODY causing mutations in *BLK* were identified in three families including p.A71T [37]. However, p.A71T was detected in 52 normoglycemic subjects in a European cohort and showed weak associations with increased T2DM risk [38].

We identified 28 potentially novel MODY-causing mutations in 34 participants (~1.1% of diabetes cases), accounting for ~56% of the overall reported mutations. Twenty-three mutations were in MODY-causing genes, while five mutations were detected in potentially novel MODY-causing genes—three variants in *RFX6* and two variants in *NKX6-1* in seven subjects. Several studies have associated variants in these genes with MODY progression [4]. Notably, the transcription factor RFX6 plays a vital role in the development of endocrine pancreas and in the differentiation of islet cells, including insulin-producing cells [39]. To date, only a few potential MODY-causing variants of *RFX6* have been identified [4,5,40]. Mutations in the *RFX6* gene, for instance, c.1954C>T (p.Arg652X), have been found to reduce insulin secretion and insulin-dependent insulinotropic polypeptide responses [5,40]. Moreover, GWAS have associated variants of *RFX6* with blood glucose levels [41] and T2DM [42]. Similarly, NKX6.1 has been identified as a critical transcription factor in the early and late development of pancreatic *β*-cells and in maintaining *β*-cells’ identity, and it regulates many genes involved in glucose uptake, metabolism and insulin biosynthesis [43]. Moreover, variants in *NKX6-1* have also been associated with T2DM [44]. Notably, only two mutations in *NKX6-1*, P329L and S317L have been described in South India and associated with MODY development [4]. These genes could have a profound effect on MODY onset, but further genetic and functional investigations are warranted.

Despite recent advances in technology, the genetic and molecular basis of MODY onset remains to be fully elucidated. The complexity and broad spectrum of phenotypes associated with MODY, including de novo mutations and genetic background, make it challenging to conduct a clinical manifestation profile for all subtypes. However, identification of population-specific mutations attributed to MODY has the potential for the development of diagnostic or prognostic microarrays as a more feasible option. For instance, multiplex ligation-dependent probe amplification led to the identification of two de novo mutations (deletions) in the *HNF1B* gene in suspected MODY cases in a Greek cohort [45]. 

Our findings highlight the genetic anomalies associated with MODY in Qatar based on a substantially large cohort. Importantly, our findings also reiterate the importance of genetic testing for MODY diagnosis given that our cohort predominantly comprised subjects classified as overweight or obese. Moreover, of the 101 suspected MODY subjects who carried mutations in MODY-related genes, 84 patients (83.2%) were overweight or obese, consistent with high obesity prevalence in Qatar. Therefore, the widely accepted clinical criteria for MODY diagnosis is not accurate when applied to our study cohort given that most patients were overweight or obese, and genetic testing is critical in MODY diagnosis.

Our study was, however, limited by the lack of detection for structural variants of MODY-associated genes, although MODY is predominantly caused by missense mutations. Moreover, additional clinicopathological data to investigate associations between specific mutations and disease severity, age of diagnosis and mode of inheritance (inherited or de novo) were not available. In addition, details about autoantibody detection to stratify patients into diabetes subtypes were also unavailable. Overall, the reported variants and mutations may be explored further in functional analysis to confirm their pathogenicity in other populations and in the development of diagnostic and prognostic tools for MODY.

## 4. Materials and Methods

### 4.1. Study Participants

Qatar Biobank (QBB) was the primary source of study participants. QBB aims to gather information from the native Qatari population, as well as from long-term residents (≥15 years), with follow-ups scheduled every 5 years. The study cohort analyzed in this study consisted of 14,364 Qatari participants aged between 18 to 89 years. Informed consent was obtained from all participants prior to inclusion in the study. The study was approved by the institutional review boards of QBB (E-2019-QF-QBB-RES-ACC-0179-0104) and Hamad Bin Khalifa University (approval no. 2021-03-078). Participants underwent extensive medical examination, filled in a standardized questionnaire and self-reported past medical histories, diet, physical activity and lifestyle [26]. Physical measurements, such as the body mass index (BMI), and biological samples, including blood, urine and saliva, were also collected. Measurements relevant to diabetes were investigated by the Qatar National Health Service (Hamad Medical Corporation, Doha, Qatar) to assess the participants’ health status by evaluating clinical diagnostic biomarkers, including glucose, hemoglobin A1c (HbA1c) and C-peptide levels.

### 4.2. Phenotypic Data and Patient Classification

Study participants were identified as diabetes or non-diabetes based on blood biomarkers (C-peptide, HbA1c, glucose), self-reported diabetes and self-reported administration of anti-diabetes medications. Serum C-peptide levels were determined by the sandwich electrochemiluminescence immunoassay using Elecsys C-Peptide kit (Roche, Basel, Switzerland), while HbA1c in blood was measured using turbidimetric inhibition immunoassay (TINIA) utilizing Tina-quant HbA1c Gen. 3 kit (Roche) on hemolyzed blood samples and random glucose levels in serum were measured using the enzymatic reference method with GLUC3 glucose hexokinase kit (Roche), utilizing a COBAS instrument (Roche).

We classified our study cohort based on the phenotypic data provided by QBB. We first identified T1DM as self-declared diabetes subjects receiving insulin treatment exclusively and with serum C-peptide levels < 0.5 ng/mL. Subjects who did not meet the criteria for T1DM were classified as T2DM if they met any of the following criteria: self-declared diabetes, self-reported diabetes medication or with HbA1c > 6.5%. Subjects who did not meet the T1DM or T2DM criteria were classified as non-diabetes subjects.

### 4.3. Whole-Genome Sequencing

The WGS data of the QBB study participants was provided by the Qatar Genome Project (QGP) [46]. Details of WGS and quality control measures have been described previously [47]. Briefly, genomic DNA from peripheral blood samples was extracted using Qiagen MIDI kit (Qiagen, Hilden, Germany), following the manufacturer’s protocol and using the automated QIASymphony SP instrument (Qiagen). DNA integrity was evaluated using Caliper Labchip GXII (Perkin Elmer, Waltham, MA, USA) Genomic DNA assay and was quantified using the Quant-iT dsDNA Assay (Invitrogen, Waltham, MA, USA). Whole-genome libraries were prepared using Illumina TruSeq DNA Nano kit (Illumina, San Diego, CA, USA). HiSeq X Ten (Illumina) was used to sequence genomic libraries to achieve a minimum average coverage of 30×. Library construction and sequencing was performed at Sidra Clinical Genomics Laboratory Sequencing Facility (Sidra Medicine, Doha, Qatar). The generated files were subjected to quality control using FastQC (v0.11.2) and reads were aligned to the CRCh38 reference genome. Quality control on mapped reads was performed using Picard (v1.117). A combined variant call file (gVCF) was generated for all study subjects, which contained all genetic variations detected in the QBB study participants.

### 4.4. Bioinformatics Analysis to Identify MODY-Causing Mutations

Genetic variants of the following known MODY-related genes—*HNF4A*, *GCK*, *HNF1A*, *PDX1*, *HNF1B*, *NEUROD1*, *KLF11*, *CEL*, *PAX4*, *INS*, *BLK*, *ABCC8*, *KCNJ11*, *APPL1*, *RFX6* and *NKX6-1*—were extracted from the gVCF file, based on genomic coordinates obtained from the University of California Santa Cruz (UCSC) Genome Browser [48]. We included genetic variants with minor allele frequency < 0.001 and mean sequencing depth > 20×. The average sequencing depth of MODY-related variants was 32.1×. We extracted genetic variants based on their location in each of the MODY-causing genes and related the genotype data to the clinical phenotype data for stratification into MODY subtypes. Variant annotations were performed using Human Gene Mutation Databases (HGMD) [49] to identify previously reported disease-causing mutations (DM) or likely disease-causing mutations (DM?). The clinical significance of variants was investigated using the QIAGEN Clinical Insight (Qiagen) and ClinVar database [50]. Potentially novel MODY-related mutations were determined based on a Combined Annotation-Dependent Depletion (CADD) [51] Phred score > 20 and complete disease penetrance (100%), implying that the subject had diabetes and the genetic variant showed a CADD Phred score > 20 (predicated to be among the top 1% most deleterious mutations in the human genome). Penetrance for each variant was calculated by dividing the number of carriers showing the phenotype (T1DM or T2DM as described above) by the overall number of mutation carriers (N). The functional annotations of the identified variants was performed using Sorting Intolerant From Tolerant (SIFT) [52] and Polymorphism Phenotyping v2 (Polyphen-2) [53] tools.

### 4.5. Statistical Analysis

Statistical analyses were performed using GraphPad Prism 9 software (GraphPad Software, San Diego, CA, USA). Shapiro–Wilk normality test was carried out to determine data distribution. Unpaired t-test was used for data comparisons between groups on normally distributed data, while Mann–Whitney test was used for datasets that did not show normal distribution. Chi-square (χ^2^) test was performed to determine the differences between binary variables within study cohorts. A *p* value of < 0.05 was considered statistically significant. Data are presented as mean ± standard deviation (SD).

## Figures and Tables

**Table 1:** Clinical characteristics of study cohort.

	Type 1 Diabetes	Type 2 Diabetes	Non-Diabetes
Number of subjects ^♦^	72 (0.5%)	2915 (20.3%)	11,377 (79.2%)
Age (mean ± SD)	38.43 ± 14.7 *	51.76 ± 11.8 *	35.05 ± 11.7
Male	34 (47.2%)	1216 (41.7%)	5104 (44.9%)
Female	38 (52.8%)	1699 (58.3%)	6273 (55.1%)
BMI (kg/m²) **	28.6 ± 5.1	32.3 ± 6.0 *	28.9 ± 6.0
Underweight	-	3 (0.1%)	262 (2.3%)
Normal weight	18 (25.0%)	236 (8.1%) *	2672 (23.5%)
Overweight	28 (38.9%)	889 (30.5%) *	4047 (35.6%)
Obese	25 (34.7%)	1777 (61.0%) *	4385 (38.5%)
N/A	1 (1.4%)	10 (0.3%)	11 (0.1%)
HbA1c (%)	8.8 ± 1.5 *	7.4 ± 1.8 *	5.3 ± 0.4
C-peptide (ng/mL)	0.32 ± 0.14 *	2.83 ± 1.67 *	2.02 ± 1.4
Family History of Diabetes			
Father	33 (45.8%) *	1357 (46.5%) *	4790 (42.1%)
Mother	33 (45.8%) *	1810 (62.1%) *	5113 (44.9%)
Both	18 (25.0%) *	966 (33.1) *	2504 (22.0%)
Treatment			
Insulin	72 (100.0%)	645 (22.1%)	-
Tablets	-	2011 (69.0%)	-

^♦^ Study cohort comprised of 14,364 subjects. * Statistically significant (*p* < 0.001) compared to non-diabetes controls. BMI: body mass index. ** Subjects with BMI below 18.5 kg/m^2^ were classified as underweight, between 18.5 to 24.9 kg/m^2^ as normal weight, between 25 to 29.9 kg/m^2^ as overweight and greater than or equal to 30 kg/m^2^ as obese [27]. N/A, not available.

**Table 2:** Identification of previously reported pathogenic and likely pathogenic MODY-causing mutations.

Gene *	MODY	ID	Ref/Alt	Protein Change	N	MAF	Penetrance (%)	Diagnosis (*n*)	CADD	HGMD	QCI	ClinVar	SIFT	PolyPhen-2
*HNF4A*	1	rs1555817727	C/T	p.Arg312cys	1	0.000035	100	T2DM	30	DM	P	LP	D	P.D
*GCK*	2	rs1375656631	C/T	p.Ala259Thr	3	0.000104	66.6	N.D (1) T2DM (2)	24.4	DM	P	P	D	Benign
*GCK*	2	rs104894005	C/G	p.Glu279Gln	1	0.000035	0	N.D	22.2	DM	LP	P	T	Benign
*HNF1A*	3	rs137853238	G/A	p.Arg272his	1	0.000035	100	T2DM	31	DM	P	P	D	P.D
*HNF1A*	3	rs587778397	C/T	p.Arg177Trp	5	0.000174	20	N.D (4) T2DM (1)	25.7	DM	LP	VUS	T	P.D
*HNF1A*	3	rs371717826	C/G	p.Pro379arg	3	0.000104	0	N.D (3)	26.7	DM	P	N/A	D	P.D
*HNF1A*	3	rs754729248	C/G	p.Pro379Ala	1	0.000035	0	N.D (1)	25	DM	VUS	P	D	P.D
*KLF11*	7	rs121912645	G/T	p.Ala347Ser	1	0.000035	0	N.D (1)	< 10	DM	P	P	T	Benign
*BLK*	11	rs766934515	T/G	p.Val113Gly	5	0.000174	0	N.D (5)	29.9	DM?	LP	N/A	D	P.D
*ABCC8*	12	rs761862121	T/G	p.Lys889Thr	3	0.000104	0	N.D (3)	27.5	DM	P	VUS	D	P.D

* Identified variants are reported as disease-causing (DM) or likely disease-causing (DM?) in the Human Gene Mutation Database (HGMD) and classified as pathogenic (P) or likely pathogenic (LP) in Qiagen Clinical Insight (QCI) and/or ClinVar databases. Penetrance is shown as percentage of mutation carriers with diabetes. Diagnosis is based on phenotype data. Ref, reference allele; Alt, alternative allele; N, number of heterozygous subjects; MAF, minor allele frequency; T2DM, type 2 diabetes; N.D, nondiabetes; CADD, Combined Annotation-Dependent Depletion Phred score; QCI, QIAGEN Clinical Insight classification based on the 2015/2016 ACMG/AMP Guidelines; VUS, variant of uncertain significance; D, deleterious; T, tolerated; P.D, probably damaging; N/A, not available.

**Table 3:** Identification of previously reported disease-causing or likely disease-causing variants in the Human Gene Mutation Database, predicted to be among the top 1% most deleterious mutations in the human genome.

Gene	MODY	ID	Ref/Alt	Protein Change	N	MAF	Penetrance (%) *	Diagnosis (*n*)	CADD	HGMD	QCI	ClinVar	SIFT	PolyPhen-2
*HNF4A*	1	rs769007443	G/A	p.Gly64Arg	1	0.000035	0	N.D	25.9	DM	VUS	N/A	D	P.D
*HNF4A*	1	rs371124358	G/A	p.Arg310Gln	1	0.000035	100	T2DM	23.5	DM	VUS	VUS	T	Benign
*HNF4A*	1	rs377151067	G/A	p.V404I	1	0.000035	100	T2DM	22.8	DM	VUS	N/A	T	Benign
*GCK*	2	rs193922285	C/A	p.Met462Ile	5	0.000174	40	N.D (3) T2DM (2)	22.9	DM	VUS	VUS	T	Benign
*HNF1A*	3	rs201095611	G/A	p.Ala161Thr	6	0.000209	16.67	N.D (5) T1DM (1)	27.3	DM	VUS	VUS	D	P.D
*HNF1A*	3	rs867576513	T/C	p.Met283Thr	6	0.000209	33.3	N.D (4) T2DM (2)	25.6	DM	VUS	N/A	D	P.D
*HNF1A*	3	rs368683806	G/A	p.Gly415Arg	1	0.000035	0	N.D	31	DM	VUS	VUS	D	P.D
*HNF1A*	3	rs577078110	C/A	p.His505Asn	2	0.000070	0	N.D (2)	26.3	DM	VUS	N/A	D	P.D
*HNF1A*	3	rs202039659	A/G	p.His514Arg	11	0.000383	27.2	N.D (8) T2DM (3)	24.8	DM	VUS	VUS	D	P.D
*PDX1*	4	rs753249965	G/A	p.Gly55Asp	3	0.000104	0	N.D (3)	23.7	DM	VUS	N/A	D	Benign
*HNF1B*	5	rs113042313	C/T	p.Gly370Ser	1	0.000035	0	N.D	21	DM	VUS	N/A	T	Benign
*BLK*	11	rs368427116	C/T	p.Thr270Met	5	0.000174	0	N.D (5)	25.4	DM	VUS	N/A	T	P.D

* Penetrance is shown as percentage of mutation carriers with diabetes. Diagnosis is based on phenotype data. Ref, reference allele; Alt, alternative allele; N, number of heterozygous subjects; MAF, minor allele frequency; T2DM, type 2 diabetes; N.D, nondiabetes; CADD, Combined Annotation-Dependent Depletion Phred score; HGMD, Human Gene Mutation Database; DM, disease-causing mutation; QCI, QIAGEN Clinical Insight classification based on the 2015/2016 ACMG/AMP Guidelines; VUS, variant of uncertain significance; N/A, not available; D, deleterious; T, tolerated; P.D, probably damaging.

**Table 4:** Discovery of potentially novel fully penetrant MODY-causing mutations in the Qatar Biobank (QBB) cohort.

Gene	MODY	Chr:Pos	ID	Ref/ Alt	Protein Change	N *	MAF	CADD	gnomAD MAF	SIFT	PolyPhen-2
*HNF4A*	1	20:44418486	-	T/C	p.Met237Thr	1	0.000035	26.2	-	D	P.D
*HNF4A*	1	20:44424203	rs776656815	C/G	p.Leu360Val	1	0.000035	22.8	0.0000239	D	Benign
*GCK*	2	7:44145572	-	A/T	p.Met393Lys	1	0.000035	26.2	-	D	Benign
*HNF1A*	3	12:120993541	-	G/A	p.Gly183Glu	1	0.000035	26.1	-	D	P.D
*HNF1A*	3	12:120979082	-	A/T	p.Glu105Val	2	0.000070	25.2	-	D	Benign
*HNF1A*	3	12:120999538	-	C/T	p.Ser591Phe	1	0.000035	24.1	-	D	P.D
*PDX1*	4	13:27924690	rs1357043267	G/C	p.Glu281Gln	3	0.000104	23.1	0.000119	D	Benign
*HNF1B*	5	17:37744622	-	G/A	p.Thr88Ile	1	0.000035	25.4	-	D	Benign
*HNF1B*	5	17:37731607	rs755951130	T/C	p.Asn345Asp	1	0.000035	22.6	0.0000081	T	Benign
*NEUROD1*	6	2:181678436	-	G/A	p.Thr142Ile	1	0.000035	28.2	-	D	P.D
*KLF11*	7	2:10046337	-	A/G	p.Asp77Gly	1	0.000035	27.9	-	D	P.D
*KLF11*	7	2:10048367	-	C/T	p.Pro344Ser	1	0.000035	23.2	-	D	P.D
*KLF11*	7	2:10046240	-	A/T	p.Met45Leu	1	0.000035	20.1	-	T	Benign
*CEL*	8	9:133070564	rs766487195	G/A	p.Gly467Arg	1	0.000035	26.1	0.0000080	D	P.D
*CEL*	8	9:133064413	rs748643667	G/A	p.Val29Met	1	0.000035	25.3	0.0000040	D	P.D
*CEL*	8	9:133066888	rs773198000	C/G	p.Ser243Arg	1	0.000035	22.9	0.0000242	D	P.D
*CEL*	8	9:133069087	-	A/G	p.Thr375Ala	1	0.000035	22.3	-	D	Benign
*BLK*	11	8:11554800	rs775313404	A/G	p.Tyr177Cys	1	0.000035	29.4	0.0000119	D	P.D
*ABCC8*	12	11:17404576	rs769818698	C/T	p.Val1165Met	2	0.000070	23.1	0.0000955	D	Benign
*ABCC8*	12	11:17427908	-	C/T	p.Gly692Glu	1	0.000035	22.7	-	T	Benign
*KCNJ11*	13	11:17387440	-	G/T	p.Gln131Lys	1	0.000035	24.2	-	T	P.D
*KCNJ11*	13	11:17387761	rs867211548	C/T	p.Val24Ile	1	0.000035	20.7	-	T	Benign
*APPL1*	14	3:57269587	-	A/T	p.Asn677Ile	1	0.000035	21.4	-	T	Benign
*RFX6*	-	6:116922098	rs1485759457	G/A	p.Val462Met	1	0.000035	26.1	-	D	P.D
*RFX6*	-	6:116927531	rs762356403	C/T	p.Ser797Leu	1	0.000035	22.9	0.0000081	D	Benign
*RFX6*	-	6:116924786	-	A/C	p.Asn558Thr	1	0.000035	22.4	-	T	Benign
*NKX6-1*	-	4:84498074	-	G/A	p.Ser52Phe	2	0.000070	24.2	-	D	Benign
*NKX6-1*	-	4:84498145	rs369821275	C/G	p.Met28Ile	2	0.000070	23.5	0.0000525	D	Benign

* All identified variants showed complete disease penetrance (100%) and mutation carriers were classified as type 2 diabetes based on phenotype data. Penetrance is shown as a percentage of mutation carriers with diabetes. Chr: Pos, Chromosome: Position of identified variant based on genome build CRCh38; Ref, reference allele; Alt, alternative allele; N, number of heterozygous subjects; MAF, minor allele frequency; CADD, Combined Annotation-Dependent Depletion Phred score; gnomAD, Genome Aggregation Database; D, deleterious; T, tolerated; P.D, probably damaging.

## Data Availability

The data analyzed in this study are subject to the following licenses/restrictions: the raw whole-genome sequence data from Qatar Biobank are protected and are not available for deposition into public databases due to data privacy laws. Access to QBB/QGP phenotype and whole-genome sequence data can be obtained through an ISO-certified protocol, which involves submitting a project request at https://www.qatarbiobank.org.qa/research/how-apply, subject to approval by the Institutional Review Board of the QBB. Requests to access these datasets should be directed to https://www.qatarbiobank.org.qa/research/how-apply.

## Supplementary Materials

The following supporting information can be downloaded at: https://www.mdpi.com/article/10.3390/ijms24010130/s1.
