# Supplementary material for: The Genetic Spectrum of Maturity-Onset Diabetes of the Young (MODY) in Qatar, a Population-Based Study
Source: Int J Mol Sci. 2022 Dec 21;24(1):130. doi: 10.3390/ijms24010130 (PMC9820507; doi:10.3390/ijms24010130)
Supplement: Supplementary file 1 [file ijms-24-00130-s001.zip › Supplementary_Figure_S1.pdf]

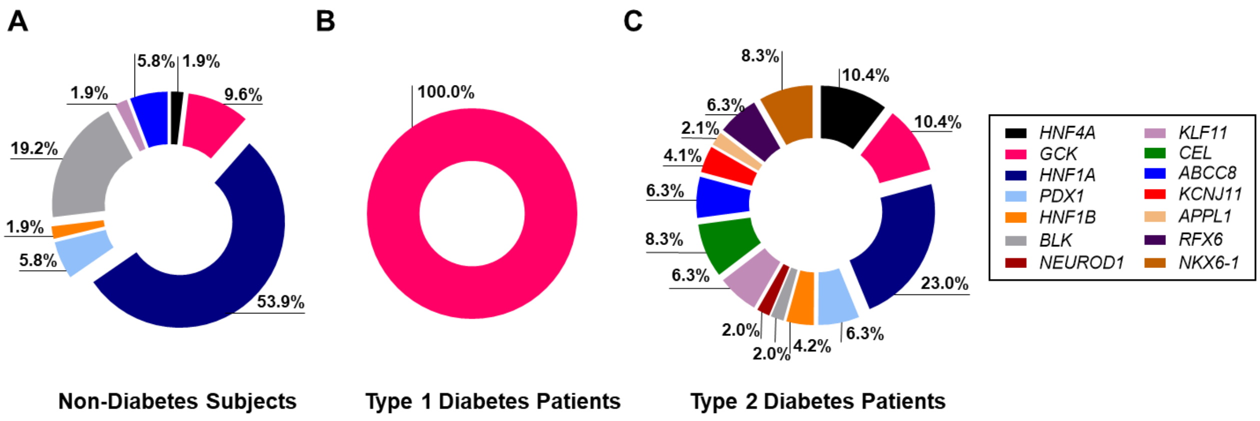

Supplementary Figure S1. Distribution of MODY- related mutations across the diabetes status categories. Donut charts represent the proportions of subjects carrying MODY-related gene mutations in **A.** Non-diabetes subjects, **B.** Type 1 Diabetes and **C.** Type 2 Diabetes subjects, classified based on clinical characteristics.
