# Supplementary material for: The Genetic Spectrum of Maturity-Onset Diabetes of the Young (MODY) in Qatar, a Population-Based Study
Source: Int J Mol Sci. 2022 Dec 21;24(1):130. doi: 10.3390/ijms24010130 (PMC9820507; doi:10.3390/ijms24010130)
Supplement: Supplementary file 1 [file ijms-24-00130-s001.zip › Supplementary_Table_S1.pdf]

Supplementary Table S1. Clinical Characteristics of MODY subjects Detected with Previously Reported MODY Mutations and Potentially Novel MODY-causing Mutations.

	**MODY cases***
Number of subjects	101
Age (mean±SD)	43.8±13.23
Male	46 (45.5%)
Female	55 (54.5)
BMI (Kg/m^2)**	
Underweight	-
Normal	17 (16.8%)
Overweight	37 (36.6%)
Obese	47 (46.5%)
Diabetic	
Yes	49 (48.5%)
No	52 (51.5%)
HbA1c (%)	6.33±1.60
C-peptide (ng/ml)	2.43±1.039
Family History of Diabetes	
Father	38 (37.6%)
Mother	58 (57.4%)
Both	27 (26.7%)
Treatment	
Insulin	13 (12.9%)
Tablets	27 (26.7%)

*MODY participants comprised of 101 subjects. BMI: body mass index. **Subjects with BMI below 18.5 kg/m^2 were classified as underweight, between 18.5 to 24.9 kg/m^2 as normal weight, between 25 to 29.9 kg/m^2 as overweight, and greater than or equal to 30 kg/m^2 as obese. N/A, not available.
