# Supplementary material for: The Genetic Spectrum of Maturity-Onset Diabetes of the Young (MODY) in Qatar, a Population-Based Study
Source: Int J Mol Sci. 2022 Dec 21;24(1):130. doi: 10.3390/ijms24010130 (PMC9820507; doi:10.3390/ijms24010130)
Supplement: Supplementary file 1 [file ijms-24-00130-s001.zip › Supplementary_Table_S2.pdf]

Supplementary Table S2. The classification (diabetes subtypes) and clinical characteristics of participants with MODY mutations.

	**Type 1 Diabetes**	**Type 2 Diabetes**	**Non-diabetes**
Number of subjects	1 (0.9%)	48 (47.5%)	52 (51.5%)
Age	26	50.0±14.2	40.9±11.4
Age <30	1 (0.9%)	6 (5.9%)	12 (11.9%)
Age ≥ 30		42 (41.6%)	40 (39.6%)
Gender			
Male	1 (0.9%)	27* (26.7%)	18 (17.8%)
Female	-	21* (20.8%)	34 (30.7%)
BMI**			
Underweight	-	-	-
Normal weight	-	3* (2.9%)	14 (13.9%)
Overweight	-	15* (14.8%)	22 (21.8%)
Obese	1 (0.9%)	30* (29.7%)	16 (15.8%)
HbA1c (%)	9.1	6.75±1.77*	5.38±0.45
C-peptide (ng/ml)	<0.08	2.6±1.19*	2.22±0.83
Genes†			
HNF4A	-	5 (4.9%)	1 (0.9%)
GCK	1 (0.9%)	5 (4.9%)	5 (4.9%)
HNF1A	-	11 (10.9%)	28 (27.7%)
PDX1	-	3 (2.9%)	3 (2.9%)
HNF1B	-	2 (1.9%)	1 (0.9%)
NEUROD1	-	1 (0.9%)	-
KLF11	-	3 (2.9%)	1 (0.9%)
CEL	-	4 (3.9%)	-
PAX4	-	-	-
INS	-	-	-
BLK	-	1 (0.9%)	10 (9.9%)
ABCC8	-	3 (2.9%)	3 (2.9%)
KCNJ11	-	2 (1.9%)	-
APPL1	-	1 (0.9%)	-
RFX6	-	3 (2.9%)	-
NKX6-1	-	4 (3.9%)	-

MODY participants comprised 101 subjects. Percentages shown in brackets are calculated from total number of MODY subjects (n=101). *Statistically significant (P < 0.001) compared to non-diabetes controls. BMI: body mass index. **Subjects with BMI below 18.5 kg/m^2 were classified as underweight, between 18.5 to 24.9 kg/m^2 as normal weight, between 25 to 29.9 kg/m^2 as overweight, and greater than or equal to 30 kg/m^2 as obese. †No. of subjects carrying MODY-gene mutation.
